# SHBG Gene Polymorphism (rs1799941) Associates with Metabolic Syndrome in Children and Adolescents

**DOI:** 10.1371/journal.pone.0116915

**Published:** 2015-02-03

**Authors:** Marquitta J. White, Fatih Eren, Deniz Agirbasli, Scott M. Williams, Mehmet Agirbasli

**Affiliations:** 1 Center for Human Genetic Research, Vanderbilt University, Nashville, Tennessee, United States of America; 2 Department of Genetics, Institute for Quantitative Biomedical Sciences, Dartmouth College, Hanover, New Hampshire, United States of America; 3 Department of Medical Biology, Marmara University School of Medicine, Istanbul, Turkey; 4 Department of Medical Biology, Acıbadem University School of Medicine, Istanbul, Turkey; 5 Department of Cardiology, Marmara University School of Medicine, Istanbul, Turkey; Children’s National Medical Center, UNITED STATES

## Abstract

**Background:**

Metabolic syndrome (MetS) is a complex disorder characterized by coexistence of several cardiometabolic (CM) factors, i.e. hyperlipidemia, obesity, high blood pressure and insulin resistance. The presence of MetS is strongly associated with increased risk of cardiovascular disease (CVD). The syndrome was originally defined as an adult disorder, but MetS has become increasingly recognized in children and adolescents.

**Methods:**

Genetic variants influence biological components common to the CM factors that comprise MetS. We investigated single locus associations between six single nucleotide polymorphisms (SNPs), previously shown to modulate lipid or sex hormone binding globulin (SHBG) levels, with MetS in a Turkish pediatric cohort (37 cases, 323 controls).

**Results:**

Logistic regression analysis revealed a significant association between rs1799941, located in SHBG, and MetS (OR = 3.09, p-value = 0.006). The association with MetS remained after sequential adjustment for each CM factor included in the syndrome definition, indicating that the identified association is not being driven by any single trait. A relationship between rs1799941 and SHBG levels, was also discovered, but it was dependent on MetS status. In control subjects, the A allele of rs1799941 associated with a significant increase in SHBG levels (p = 0.012), while in cases there was no association between rs1799941 and SHBG levels (p = 0.963).

**Conclusions:**

The significant association between rs1799941 and MetS in children is not contingent on any single CM trait. Additionally, the presence of MetS may abrogate effect of rs1799941 polymorphism on SHBG levels in children.

## Introduction

Metabolic syndrome (MetS) is a complex condition defined by the clustering of 3 or more cardiometabolic (CM) phenotypes, and is associated with increased cardiovascular disease (CVD) morbidity and mortality [1–5]. MetS, which was originally described as an adult disorder [1–3], has become increasingly recognized in children and adolescents [6]. Concurrent with the rising trend in childhood obesity, MetS has increased in prevalence in the pediatric population worldwide [7, 8]. Clustering of CM risk factors, in general, has also been shown to begin in youth [5].

Originally, MetS in children was defined as a direct consequence of childhood obesity [9]. However, CM risk traits such as high blood pressure and dyslipidemia are now common in children even in the absence of obesity [10]. Furthermore, ethnic variations exist in the distribution of CM risk traits in children [11]. In a previous study comparing children from Ankara, Turkey to the Bogalusa Heart Study population, we reported that dyslipidemia and high blood pressure were highly prevalent in children from Turkey even in the absence of obesity [12]. CM risk is also affected by sex hormone levels in children through regulation of lipids [13, 14]. Both sex hormone and sex hormone binding globulin (SHBG) levels are associated with lipid levels and insulin resistance in children and adolescents [15].

Hormonal changes at puberty lead to increased fat mass, and decreased SHBG levels [16]. Circulating SHBG is the strongest known predictor of MetS in children and adolescents, with lower SHBG levels associating with increased risk [15, 17]. Several studies present evidence of genetic variants modulating lipid levels in adult populations [18–21]. In our pediatric cohort, we previously showed that significant associations exist between SHBG (rs6257), CETP (rs708272) polymorphisms and lipid traits (high triglycerides, low HDL-C and high LDL-C) in children and adolescents [22]. Additionally, single nucleotide polymorphisms (SNPs) located in the SHBG gene (rs1799941 and rs6257) have been associated with SHBG levels [23].

In our study, we investigated several SNPs from five genes known to associate with lipid or circulating SHBG levels in adults (*LPL*, *CETP*, *LIPC*, *ABCA1*, and *SHBG*) for association with MetS in a pediatric cohort [18–23]. We also evaluated genetic variants that associate with MetS as a whole, thereby discovering underlying associations that impact only MetS, as opposed to any single trait used to define it.

## Methods

### Study population and data collection

The study (n = 360) was derived from a cross sectional survey on the prevalence of cardiovascular risk factors in a representative sample of school children in Istanbul, Turkey. Five different state elementary and secondary schools were selected. Students who did not want to participate in the survey were excluded (around 10% for elementary school children and 20% for secondary school students). Blood samples were drawn at 9 am. The Institutional Review Board of Marmara University and the Educational Board approved the study protocol. Informed consent was obtained from parents or guardians. Subjects were given case numbers and identities were kept confidential. At least one parent signed an informed consent for participation.

### Cardiometabolic risk variables and definitions

**Anthropometry.** Body mass index (BMI) was calculated as weight (kg)/height (m^2^). Using the tables provided by the waist circumference percentiles in a nationally representative sample, we determined subjects with increased waist circumference (≥ 90th percentile) [24]. Age- and sex-specific cutoff points of BMI were also used to assess the overweight and obesity status [25].

### Elevated blood pressure

Blood pressure was measured by automatic blood pressure monitor (Omron) 3 times while the subjects were seated, and the last two measurements were averaged for analysis. Small and medium-sized cuffs were used for arm circumferences of < 22 and 22–32 cm respectively. To find the age specific height percentile level for each individual, we used the growth curves drawn for healthy Turkish Children [26]. Using the tables provided by the Task Force Report on High Blood Pressure in Children and Adolescents, we determined children and adolescents with elevated blood pressure (≥ 95th percentile) [27].

### Biochemical analysis

Patients were considered to be fasting if they reported at least 12 hours of fasting prior to blood sampling. Complete lipid profiles were obtained, including total cholesterol (TC), high-density lipoprotein cholesterol (HDL-C), triglyceride (TG), low-density lipoprotein (LDL-C) (calculated with the Friedwald calculation: LDL = TC − HDL − [TG/5]). TG, HDL-C and glucose were measured by enzymatic colorimetric-assay method using Cobas Integra 800 kit (Roche Diagnostic). Insulin and sex hormone binding globulin (SHBG) levels were measured by chemiluminescence immunoassay method using Modular E170 kit (Roche Diagnostic). Analyses were performed in an accredited laboratory (Centro Laboratorıes which are based in Istanbul / Turkey).

**Hyperlipidemia and insulin resistance.** A TG level ≥ 75th percentile or HDL-C level ≤ 25th percentile were used to define high TG and low HDL, respectively [28]. Impaired fasting glucose (IFG) was defined as ≥ 100 mg/dL [29]. Hyperinsulinemia was arbitrarily defined as fasting value ≥ 18 μU/mL, a value considered indicative of insulin resistance in normoglycemic subjects [30]. Homeostasis model assessment-estimated insulin resistance (HOMA-IR) was calculated using the formula (glucose [mmol/L] x fasting insulin [μU/ml]/22.5) [31]. Insulin resistance is defined based on a threshold of ≥ 3.16 [32].

**Definitions of metabolic syndrome.** Based on the adopted NCEP criteria [1], Subjects with 3 or more CM risk criteria were considered to have MetS. The risk criteria included: elevated systolic and/or diastolic blood pressure (≥ 95th percentile), overweight or obesity status based on an age- and sex-specific cutoff points of BMI, elevated TG, low HDL-C, and impaired fasting glucose (IFG). WHO and IDF definitions of MetS were also used [2–4]. IFG or insulin resistance (assessed by HOMA-IR) and waist circumference (≥ 90 percentile based on a nationally representative sample) are the prerequisites for WHO and IDF definitions, respectively. Additionally, both definitions require the presence of at least two other CM risk factors. Subjects were assessed for all three definitions of MetS and children who fulfilled the criteria for at least one definition were classified as having MetS in the current study.

**Determination of the genotypes.** Two ml of venous blood from subjects were collected and stored at + 4°C prior to DNA isolation. The LPL stop codon polymorphism rs328 (S447Ter (terminal codon), ABCA1 promoter polymorphism rs1800977 (C – 14T), LIPC promoter polymorphism rs1800588 (C – 514T), and CETP promoter polymorphism rs708272 were analyzed—using restriction fragment length polymorphism (RFLP) after being amplified by polymerase chain reactions (PCR). A base substitution from G (B1) to A (B2) in intron 1 of the CETP gene leads to 3 genotypes, B1B1, B1B2 or B2B2 at Taq1B site (5454G>A). RFLP genotyping of the CETP polymorphism was carried out as previously described [33]. For SHBG polymorphisms, rs1799941 and rs6257, genotyping was performed by real time PCR amplification and fluorescent probe melting point analysis on the Light Cycler 1.5 instrument (Roche Diagnostics GmbH, Mannheim, Germany) (S1 Table). For these PCR reactions, 50 ng genomic DNA was amplified with the Light Cycler Fast Start DNA Master HybProbe Kit (Roche Diagnostics GmbH, Mannheim, Germany). Each 20μl reaction contained 1X Fast Start DNA Master HybProbe, 1.5mM MgCl2, 0.3μM each primer and 0.15μM hybridization probe. After initial denaturation step at 95°C for 10 minutes, amplification was performed using 45 cycles of denaturation at 95°C for 5s, annealing at 55°C for 20s and extension at 72°C for 20s. This was followed by a melting curve analysis of 95°C for 0 s, 40°C for 30s and a slow ramp (0.1°C /second) to 85°C with continuous fluorescent acquisition.

**Statistical Analyses.** All marker genotypic distributions were assessed for deviations from Hardy Weinberg Equilibrium (HWE) in the full dataset, and in cases and controls separately, (S2 Table). HWE was tested using the software package PLINK [34]. A HWE p < 0.001 was used to indicate significant deviation from equilibrium. Continuous and dichotomous demographic variables were assessed for significant differences between MetS cases and controls prior to SNP genotype-phenotype analyses. For continuous variables the Shapiro-Wilkes test was applied in order to determine if the variable was normally distributed. In instances where a variable was not normally distributed (Shapiro Wilkes p < 0.05), and standard statistical tests would be inappropriate, non-parametric analytical techniques were used. Unless otherwise indicated, all statistical analyses in this study were performed, using the statistical package STATA 11 [35].

**Preliminary Association Testing.** Logistic regression analysis was used to identify and quantify associations between SNPs and MetS. Logistic regression models were adjusted for age, gender, and circulating SHBG levels. Due to small sample size, dominant coding was used for SNP genotype with the homozygous major genotype as the referent and the heterozygote and homozygous minor genotypes were collapsed into one category (S3 Table). A nominal p-value < 0.05 was used to signify a significant association and Bonferroni correction was applied to account for multiple testing.

**Confirmation of the Integrity of Identified Association(s).** Once a significant association was revealed in preliminary analyses, we tested for the integrity of the genotype-phenotype relationship by creating several independent adjusted models that included the nominally associated SNPs, covariates (age, gender, and circulating SHBG levels), and the six major defining factors of MetS: low HDL-C, high TG, increased waist circumference, insulin resistance, obesity, and elevated blood pressure in a step-wise fashion. These additional models were compared to the reference model that contained only SNP, age, gender, and circulating SHBG levels. Each variable was dichotomized according to preset definitions prior to inclusion in the adjusted analyses (S4 Table). Loss of significance in these models (p ≥ 0.05) was used to identify confounded associations between SNP(s) and MetS.

**Assessment of the effect of SNPs in SHBG on circulating SHBG levels.** Due to the non-normality of circulating SHBG, we applied two complimentary non-parametric methods, the Kruskal-Wallis (K-W), and the Non-Parametric (NP) Trend Test to investigate the difference in SHBG levels as a function of SNP genotype. The K-W test is an alternative to the standard Analysis of Variance (ANOVA) analysis and tests the null hypothesis of no difference in SHBG levels between SNP genotype groups. For the NP Trend test, SNP genotypes were ordered by number of minor allele (0 = homozygous major, 1 = heterozygote, 2 = homozygous minor). Arranged in this manner, the NP Trend test assessed whether there was an increasing trend in circulating SHBG levels for each additional copy of the minor allele. A p-value < 0.05 was used to determine significance of K-W and NP analyses.

In order to determine the direction of effect for significant associations identified from the K-W and/or NP Trend tests, median regression was performed. To thoroughly explore the effect of SNP genotype on SHBG levels two separate SNP genotype coding schemes, additive (coded 0, 1, or 2, depending on the number of minor alleles) and dominant (coded 0 or 1 in reference to the absence or presence of any minor allele), were implemented, in order to determine if there was a difference of effect between case and control subjects. All median regression models were adjusted for age and gender, and significance threshold was set at p < 0.05. Visual representations of the relationship between median or mean circulating SHBG and SNP genotype were created, using version 2.11.3 of the gplots package, in the R statistical platform [36, 37].

## Results

A subset of 360 Turkish children and adolescents (170 males, 190 females) was extracted from a previously described cohort (n = 365) collected by Agirbasli et al [15]. All individuals with available DNA samples from the original cohort were included in the present study. Demographics of all analyzed individuals are presented in Table 1.

**Table 1:** Distribution of Demographics and Circulating Sex Hormone Binding Globin Levels in Metabolic Syndrome Cases and Controls.

Metabolic Syndrome Risk Factors	Metabolic Syndrome Cases (n = 37)	Controls (n = 323)	P-Value
Gender^1^	29.7%	55.4%	0.003^▪^
Age^2^	12.2 (0.54)	13.1 (0.19)	0.020^◊^
Family History of Cardiovascular Disease (CVD)^3^	[26(9)]	[200(103)]	0.325
Family History of Hyperlipidemia (LPD)^4^	[29(6)]	[212(90)]	0.116
Parental Smoking (PS)^5^	[14(23)]	[133(190)]	0.696
Sex Hormone Binding Globin (SHBG)^6^	57.6 (7.16)	75.3 (2.38)	0.005^◊^

*Note*: P-Values presented above are from the Chi-Square test of Association, unless otherwise indicated.

^1^ Percentage of indicated Metabolic Syndrome subset (i.e. cases or controls) that is female

^2^ Mean (Standard Error) of age measured in years

^3^ CM: [CVD Controls (CVD Cases)], CVD Case definition is presence of family history of Cardiovascular Disease

^4^ LPD: [LPD Controls (LPD Cases)], LPD Case definition is presence of family history of dyslipidemia

^5^ PS: [PS Cases (PS Controls)], PS Case definition is presence of either maternal or paternal smoking in the participant’s primary residence

^6^Mean (Standard Error) of Sex hormone binding globulin measured in nmol/l

^▪^P-value presented is from the two group test of proportions in STATA 11

^◊^P-value presented is from Wilcoxon Rank Sum test

### Description of Single Nucleotide Polymorphisms

The chromosomal location, allele definitions, genotype distribution, and Hardy-Weinberg equilibrium (HWE) measurements are presented in S1 and S2 Tables. No genotyped markers deviated significantly from HWE in the full dataset, or in case/control subsets (all p > 0.001).

### Dichotomous analysis of Metabolic Syndrome by genotype groups

A total of 37 subjects (11 males, 26 females) met at least one of the three established definitions of MetS and were uniformly defined as MetS cases, regardless of the number of MetS definitions (WHO, NCEP, IDF) that identified them (Table 2, S5 Table). The remaining 323 individuals in the study cohort were designated controls. Logistic regression analysis was performed to identify significant associations between genotype and MetS for all analyzed SNPs (Table 3). The models, dominantly coded, were adjusted for age, sex, and circulating SHBG levels. Having at least one minor allele (A allele) at rs1799941, located in *SHBG*, associated with increased odds of MetS (OR: 3.09, p = 0.006) and the effect remained after correction for multiple testing (Table 3, S6–S7 Tables). None of the other analyzed markers associated with MetS.

**Table 2:** Metabolic Syndrome Case definition.

Metabolic Syndrome Definition Source	Metabolic Syndrome Definition Criterion	Cases Identified^1^
Adopted National Cholesterol Education Program (NCEP)	Presence of at least THREE of the following:	33
	1. Elevated diastolic or systolic blood pressure (> 95^th^ percentile)	
	2. Overweight or obese (BMI)	
	3. Elevated triglycerides (> 75^th^ percentile)	
	4. Low HDL cholesterol (<25^th^ percentile)	
	5. Elevated Glucose (> 100mg/dL)	
World Health Organization (WHO)	Impaired fasting glucose (glucose > 100mg) or Insulin Resistance (HOMA-IR > 3.16) AND at least TWO of the following:	9
	1. Elevated blood pressure	
	2. Overweight or Obese (BMI)	
	3. Elevated triglycerides (>75^th^ percentile)	
	4. Low HDL cholesterol (<25^th^ percentile)	
International Diabetes Foundation (IDF)	Increased waist circumference (≥ 90^th^ percentile; ethnicity specific) AND at least TWO of the following	5
	1. Raised triglycerides (≥ 150 mg/dl	
	2. Reduced HDL-cholesterol (≤40mg/dL for boys, ≤ 50mg/dL for girls)	
	3. Raised blood pressure (systolic ≥ 130 mm Hg, diastolic ≥85 mm Hg)	
	4. Elevated fasting glucose (>100 mg/dL)	
Current Metabolic Syndrome Study	Subjects that fulfill the criteria for at least ONE of the three metabolic syndrome definitions: Adopted NCEP, WHO, IDF	37^2^

*Note*: Where applicable, above definitions show adjustments from adult thresholds to better fit a pediatric cohort; for ethnicity specific thresholds, the Turkish population was used as a reference unless otherwise indicated.

^1^Number of metabolic syndrome cases identified according to the specified definition; subjects may fulfill the criteria for multiple definitions simultaneously.

^2^Number represents unique cases that fulfill the criteria presented by at least one of the following sources: Adopted NCEP, WHO, or IDF. This is the number of cases used in the present study.

**Table 3:** Logistic regression results of Metabolic Syndrome Adjusted for known Metabolic Syndrome Risk Factors.

GENE	SNP	OR^1^	95% Confidence Interval	P-Value
ABCA1	rs1800977	1.61	0.76–3.43	0.216
LPL	rs328	0.92	0.36–2.32	0.859
CETP	rs708272	0.67	0.31–1.45	0.312
LIPC	rs1800588	1.01	0.47–2.16	0.988
SHBG	rs1799941	3.09	1.38–6.91	0.006*
	rs6257	0.93	0.43–1.97	0.841

*Regression models above were adjusted for age*, *gender*, *and SHBG levels*. *Significant results are highlighted in****bold***.

*Note*: SNPs were coded coded dominantly for the effect of the minor allele (homozygous major = 0, heterozygote = 1, homozygous minor = 1). Odds ratios presented above represent the change in odds per the addition of at least one copy of the minor allele for each analyzed SNP.

^1^: OR = Odds Ratio

*Effect remained significant after Bonferronni correction for multiple testing

Identification of genetic variants that associate with MetS, but not with any single factor, supports the concept of MetS as a legitimate cohesive phenotype. Associations with MetS that lose significance after accounting for the effect of a single factor would support the conclusion that MetS is not a syndrome but merely the collection of unrelated independent traits. We constructed a series of logistic regression models that sequentially adjusted for the inclusion of single CM traits (S4 Table), and to assess effect on the association between rs1799941 and MetS (Table 4). The association between rs1799941 and MetS remained significant (2.67 < ORs < 4.22; 0.003 < p-values < 0.028) after adjustment for each CM factor included in the MetS definition, confirming an association between rs1799941 and MetS.

**Table 4:** Behavior of the association between rs1799941 and metabolic syndrome when the effects of single biological features defining metabolic syndrome are held constant.

		rs1799941 (*SHBG*)		
Model Type^1^	Model Composition	OR^2^	95% CI^3^	P-value^4^
Unadjusted	rs1799941, age, gender, SHBG levels	3.09	1.38–6.91	**0.006**
Adjusted (High TG)	rs1799941, age, gender, SHBG levels, High TG	3.10	1.13–8.52	**0.028**
Adjusted (Low HDLC)	rs1799941, age, gender, SHBG levels, Low HDLC	4.22	1.48–12.04	**0.007**
Adjusted (Obese/Overweight)	rs1799941, age, gender, SHBG levels, Obese/Overweight	3.80	1.47–9.83	**0.006**
Adjusted (Elevated BP)	rs1799941k age, gender, SHBG levels, Elevated BP	3.69	1.57–8.67	**0.003**
Adjusted (Insulin Resistance)	rs1799941, age, gender, SHBG levels, insulin resistance	2.89	1.28–6.54	**0.011**
Adjusted (Waist Circumference)	rs1799941, age, gender, SHBG levels, Waist Circumference	2.67	1.09–6.49	**0.031**

*Significant results are highlighed above in****bold***.

*Note*: *rs1799941 was coded dominantly for the effect of the minor allele (homozygous major = 0*, *heterozygote = 1*, *homozygous minor = 1)*. *Odds ratios presented above represent the change in odds per the addition of at least one copy of the minor allele*. *All defining Metabolic syndrome criteria with sufficient sample size (n≥5) were evaluated above*.

^1^ Indicates the specific logisitic regression model tested: Unadjusted, or Adjusted (Possible Confounder included in model).

^2^ Indicates odds ratio (OR) for logisitc regression analysis between rs1799941 and metabolic syndrome in the specified model.

^3^ Indicates the 95% Confidence Interval associated with the presented odds ratio for each specified model.

^4^ Indates the p-value associated with the presented odds ratio for each specified model.

### Investigation of circulating SHBG as a possible association intermediary

**Fig 1:**
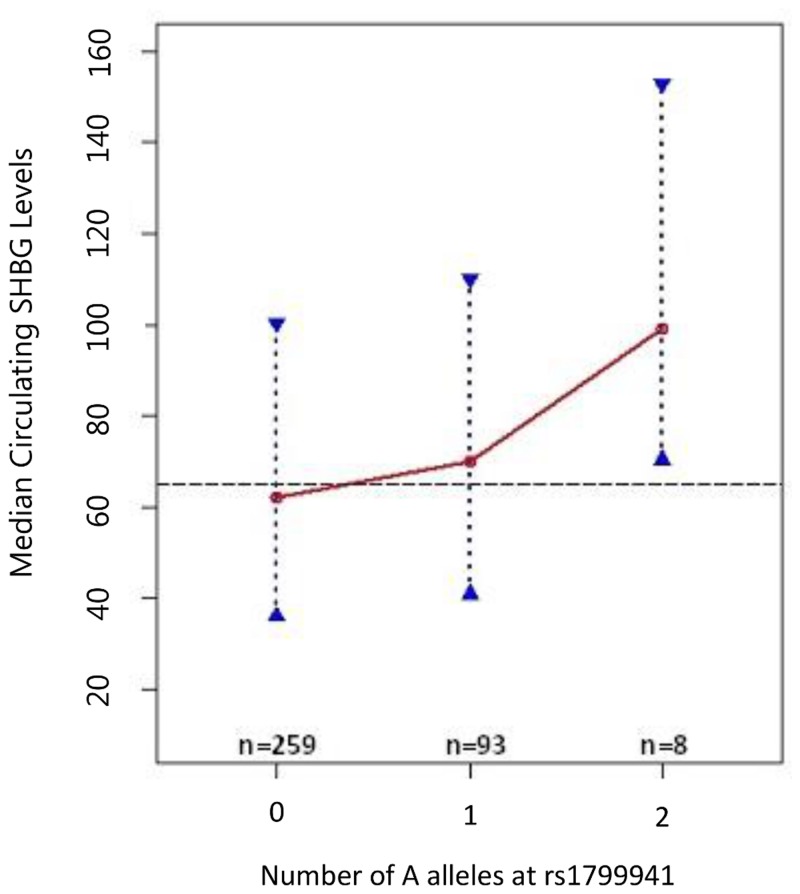
Median Circulating Sex Hormone Binding Globulin Levels vs. rs1799941 genotype (additive coding). Fig. 1 presents the relationship between median SHBG levels and rs1799941 coded additively for increasing numbers of the minor allele (A allele). Dashed line represents overall median SHBG level (65.0 nmol/l) in full cohort. Red circles represent denoted genotypic medians, and blue dotted lines with triangular end bars represent the interquartile range for SHBG in the rs1799941 genotype subgroup indicated. Connecting red lines illustrate trend in median SHBG by rs179941 genotype.

Due to the genic location of rs1799941 in *SHBG*, we investigated whether circulating SHBG modulates the SNP’s association with MetS. We employed non-parametric tests, Kruskal-Wallis (K-W) and Non-Parametric Trend Test (NP), to determine whether a significant relationship existed between rs1799941 and circulating SHBG levels. Our results demonstrated that circulating SHBG levels differed significantly by genotype (K-W p-value = 0.049), and furthermore, that the minor allele correlated with an increase in SHBG levels (NP p-value = 0.031) (Table 5). To determine the effect size of the association identified by K-W and NP analyses, we performed median regression with rs1799941 genotype coded both additively and dominantly, adjusting for age and gender. Median regression results showed that for the additive model there was an eight nmol/l increase (p = 0.016), while for the dominant model we observed a seven nmol/l (p = 0.024) in median SHBG (Table 6, Fig. 1).

**Table 5:** Sex Hormone Binding Globulin Levels (SHBG) by rs1799941 genotype.

	rs1799941 genotype groups			Tests used to assess differences in SHBG between genotype groups	
SHBG	GG	AG	AA	K-W P-value ^1^	NP Trend P-Value ^2^
Median (IQR)	62.00 (36–101)	70.00 (41–110)	99.00 (70.5–153)	**0.049**	**0.031**
Mean (SE)	70.89(2.61)	77.88(4.674)	106.88(16.80)		

*Significant results are highlighted in****bold***.

*Note*: The above analyses were performed in the combined Full Cohort, without regard to Metabolic Syndrome status.

^1^P-value presented is for the Kruskal Wallis test for differences between groups

^2^P-value presented is from the Non-Parametric Trend test (increasing trend)

**Table 6:** Regression Analysis of Sex Hormone Binding Globulin Levels by rs1799941 genotype.

	Linear Median Regression			
rs1799941 Genotype Coding ^*^	Coef.^3a^	SE^4^	P-value	95% CI^5^
rs1799941_ADD^1^	8.00	3.30	**0.016**	1.50–14.50
rs1799941_DOM^2^	7.00	3.10	**0.024**	0.91–13.09

*All regression models were adjusted for age and gender*. *Significant results are highlighted in****bold***.

*Describes the coding scheme used for rs1799941

^1^ rs1799941 genotype was coded dominantly with the minor allele as the reference: homozygous major = 0, heterozygote = 1, homozygous minor = 1

^2^ rs1799941 genotype was coded additively with the minor allele as reference: homozygous major = 0, heterozygote = 1, homozygous minor = 2

^3a^ Regresssion coefficient that describes the change in median SHBG levels per the addition of one (rs1799941_ADD) or at least one (rs1799941_DOM) minor allele at SNP rs1799941

^4^ Standard Error of regression coefficient

^5^ 95% Confidence Interval for indicated regression coeficient

**Fig 2:**
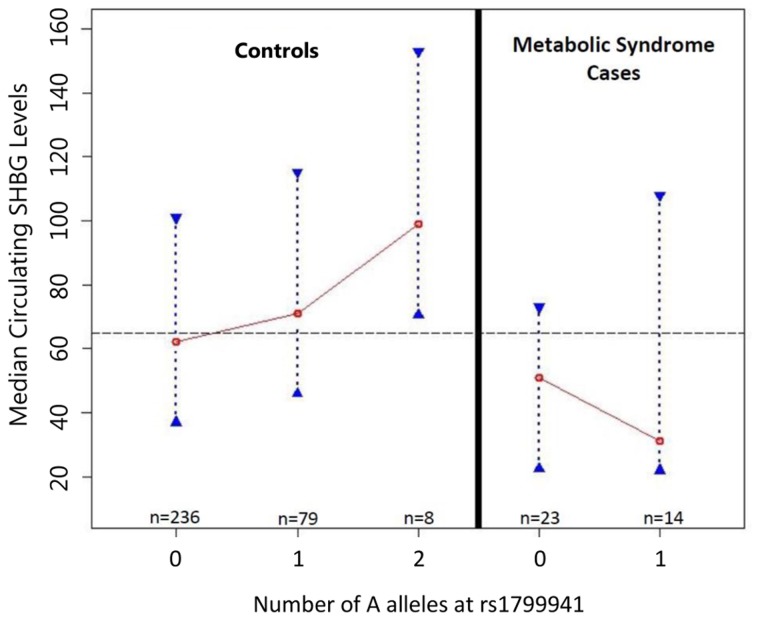
Median Circulating Sex Hormone Binding Globulin Levels vs. rs1799941 genotype (dominant coding). Fig. 2 presents the relationship between median SHBG levels and rs1799941 coded dominantly for increasing numbers of the minor allele (A allele). Dashed line represents overall median SHBG level (65.0 nmol/l) in full cohort. Red circles represent denoted genotypic medians, and blue dotted lines with triangular end bars represent the interquartile range for SHBG in the rs1799941 genotype subgroup indicated. Connecting red lines illustrate trend in median SHBG by rs179941 genotype.

**Fig 3:**
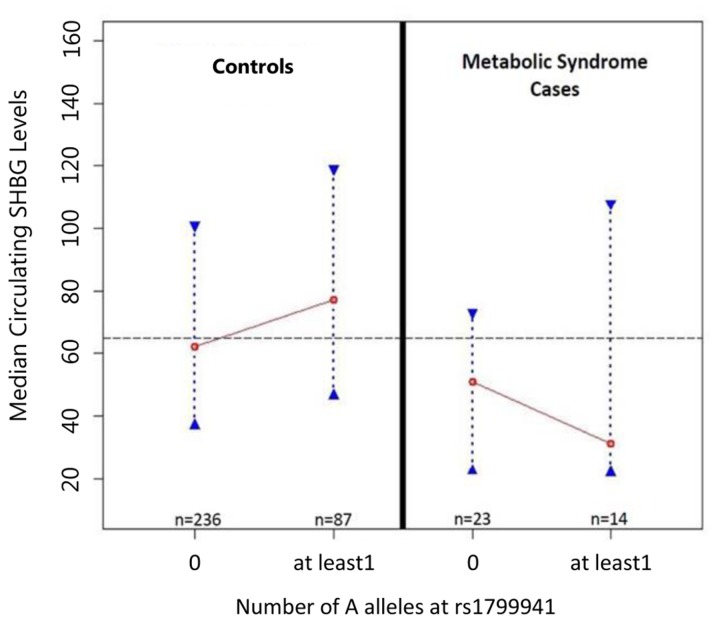
MetS status and the relationship between rs1799941 and SHBG. Fig. 3 assesses whether MetS status affected the relationship between rs1799941 and SHBG. The association with increased circulating SHBG levels in the full dataset was driven by control subjects only (K-W p-value = 0.031, NP p-value = 0.012).

The minor allele of rs1799941 associated with both increased odds of MetS and increased circulating SHBG levels. Since previous studies indicated that higher SHBG associated with a decrease in risk of MetS [15], we assessed whether MetS status affected the relationship between rs1799941 and SHBG. Results from our MetS status stratified analyses showed that the association with increased circulating SHBG levels in the full dataset was driven by control subjects only (K-W p-value = 0.031, NP p-value = 0.012) (Table 7, Figs. 2–3). Because of the significant differences in the distributions of age and gender between MetS cases and controls (Table 1, S8 Table), we performed explicit tests to evaluate the validity of the observed difference in median/mean SHBG seen between MetS cases and controls when we adjusted for the effects of age and gender. Median regression analyses revealed that after accounting for the effects of age and gender, median SHBG levels were still significantly associated with MetS status (p = 6.48E-09) (S9–10 Tables).

**Table 7:** Sex Hormone Binding Globulin Levels (SHBG) by rs1799941 genotype in Metabolic Syndrome Cases and Controls.

		rs1799941 genotype groups			Non-Parametric Tests to assess differences in SHBG levels between genotype groups	
	SHBG	GG	AG	AA	K-W P-value^1^	NP Trend P-Value^2^
Controls	Median [IQR]	62 [37–101]	71 [46–115]	99 [70.5–153]	**0.031**	**0.012**
	Mean (SE)	72.18 (2.71)	81.48 (4.96)	106.88 (16.80)		
Metabolic Syndrome Cases	Median [IQR]	51 [22–74]	31 [22–108]	NA	0.963	0.963
	Mean (SE)	57.7 (9.17)	57.6 (11.91)	NA		

Significant results are highlighted in ***bold***.

NA: There were no individuals in this genotype group

^1^P-value presented is for the Kruskal Wallis test for differences between groups

^2^P-value presented is from the Non-Parametric test for Increasing Trend in STATA 11

To quantify the newly observed relationship between rs1799941 genotype and circulating SHBG levels, we performed median regression analyses, adjusting for age and gender (Table 8). In controls, after accounting for the effects of age and gender, the significant association between rs1799941 and SHBG levels remained (p-value = 0.021) (Table 8). In contrast, there was no evidence of association between rs1799941 and circulating SHBG levels in cases (p-value = 0.332) (Table 8). Although non-significant, the effect of the A allele in MetS cases trended in the opposite direction compared to controls (Figs. 2–3, S1–S2 Figs., S6–S7 Tables). Overall, these results suggest that the mechanism through which rs1799941 affects circulating SHBG levels is inhibited or impaired in the presence of MetS.

**Table 8:** Regression Analysis of Sex Hormone Binding Globulin Levels by rs1799941 genotype in Metabolic Syndrome Cases and Controls.

	rs1799941 Genotype Coding^*^	Linear Median Regression			
		Coef.^3^	SE^4^	P-value	95% CI^5^
Controls	rs1799941_ADD^1^	9.07	3.91	**0.021**	1.37–16.77
	rs1799941_DOM^2^	13.00	4.89	**0.008**	3.37–22.62
Metabolic Syndrome Cases	rs17999413^#^	14.00	14.23	0.332	-14.96–42.95

*All regression models were adjusted for age and gender*. *Significant results are highlighted in****bold***.

*Describes the coding scheme used for rs1799941 1

^1^ rs1799941 genotype was coded dominantly with the minor allele as the reference: homozygous major = 0, heterozygote = 1, homozygous minor = 1

^2^ rs1799941 genotype was coded additively with the minor allele as reference: homozygous major = 0, heterozygote = 1, homozygous minor = 2

^#^ There are no Metabolic Syndrome cases with the AA genotype; therefore, additive and dominant genotype coding in these individuals is identical

^3^ Regresssion coefficient that describes the change in median SHBG levels per the addition of one (rs1799941_ADD) or at least one (rs1799941_DOM) minor allele at SNP rs1799941

^4^ Standard Error of regression coefficient

^5^ 95% Confidence Interval for indicated regression coeficient

## Discussion

Our results indicate that rs1799941 associates significantly with MetS in children but not with any defining CM trait. Additionally, the direction of the relationship between rs1799941 genotype and circulating SHBG levels differed significantly between subjects with and without MetS. Two recently published studies using the same population provide strong evidence that circulating SHBG is involved in the pathogenesis of MetS in children and adolescents [17, 38]. Low SHBG level was a significant predictor of insulin resistance, low HDL-C and MetS in children [15, 38].

Genetic variation in *SHBG* affects the circulating sex hormone levels [23, 39]. Therefore, genetic factors that lower SHBG levels may potentially affect MetS defining traits, including high blood pressure, and obesity [40]. *SHBG* polymorphisms associate with insulin resistance [41, 42]. Previous studies report that the G allele of rs1799941 in *SHBG* associates with lower SHBG levels [40–42]. We replicate these findings, but in control subjects only.

Our findings also support those from adult populations; lower levels of SHBG associate with a higher risk of MetS as well as several CM risk components in postmenopausal women [40–42]. Additionally, allele A of rs1799941 in *SHBG* associated with higher SHBG levels and lower BMI, waist circumference, and systolic and diastolic blood pressure in postmenopausal women [42]. SHBG polymorphisms are predictive of type 2 diabetes mellitus risk in the Physicians Health Study [41]. In a study from Northern Spain both rs1799941 (A/G) and rs6257 (T/C) in SHBG showed significant associations with serum SHBG levels [43]. The G allele of the rs1799941 and the T allele of rs6257 polymorphisms of SHBG gene associated with low SHBG levels and CM risk in adult populations [42, 43]. Previous studies have shown that SHBG levels vary by ethnicity and race in children, as does MetS [44]. For example, South Asian children who had one parent with MetS presented with 24% lower SHBG as compared to controls, and 55% less if both parents had MetS [45]. Hergenc et al report that Turkish middle-aged adults had similar total testosterone but lower SHBG levels compared with Germans [46]. In the cross-sectional study, univariate analysis displayed that HDL-C had positive correlations with SHBG in both sexes. Multivariate analysis demonstrated that most of the differences in HDL-C levels between Germans and Turks were explained by ethnicity, independently of obesity markers, insulin, and SHBG levels. Lower SHBG levels may help us to understand the causes of CM risk in Turkish population [47].

Our results, demonstrating a significant association between rs1799941 and SHBG levels in controls but not MetS cases, indicate a complex regulatory milieu. These results indicate that the effects of the rs1799941 polymorphism are disease context dependent and may be affected by the presence of one or more CM phenotypes. It should be noted that due to our small sample size, and the possibility that our detected effects are population-specific, these analyses will need to be confirmed in a larger cohort and in several populations in order to demostrate the validity of our findings. Previous studies have shown that SHBG levels, as MetS, vary by ethnicity and race in children [45–47]. Therefore, these analyses will need to be repeated in several populations in order to determine the generalizability of our results. The study was a cross sectional survey of school students with several limitations. Environmental risk factors such as diet or physical activity, which we did not measure in our study, can alter protein levels, thereby modifying genetic effects. Despite these caveats, a robust genetic effect on susceptibility to MetS was detectable. The mechanism(s) through which this variant operates is as yet undefined, and is further complicated by the variable relationship between rs1799941 and SHBG levels according to MetS phenotype. These results are suggestive of a complex regulatory system that will require larger studies to assess the role of *SHBG* genotype on MetS in concert with other genetic and environmental risk factors.

## Supporting Information

S1 Fig Mean Circulating Sex Hormone Binding Globulin Levels vs. rs1799941 genotype (additive coding). S1 Fig. presents the relationship between mean SHBG levels and rs1799941 coded additively for increasing numbers of the minor allele (A allele). Dashed line represents overall mean SHBG level (73.8 nmol/l) in full cohort. Red circles represent genotypic means, and error bars represent 95% Confidence Intervals of associated genotypic means. Connecting red lines illustrate trend in mean SHBG levels by rs179941 genotype.
(TIF)

S2 Fig Mean Circulating Sex Hormone Binding Globulin Levels vs. rs1799941 genotype (dominant coding). S2 Fig. illustrates the relationship between mean SHBG levels and rs1799941 genotype coded dominantly for increasing numbers of the minor allele (A allele). Dashed line represents overall mean SHBG level (73.8 nmol/l) in full cohort. Red circles represent genotypic means, and error bars represent 95% Confidence Intervals of associated genotypic means. Connecting red lines illustrate trend in mean SHBG levels by rs179941 genotype.
(TIF)

S1 Table Genotyped single nucleotide polymorphisms (SNPs). (DOC)

S2 Table Genotype distribution and Hardy Weinberg Equilibrium (HWE) Analysis for all SNPs. (DOC)

S3 Table Dominantly Coded Genotype Distribution for all SNPs in Metabolic Syndrome Cases and Controls. (DOC)

S4 Table Definition of dichotomized cardio-metabolic traits included in adjusted logistic regression models for the evaluation of the association between rs1799941 and Metabolic Syndrome. (DOC)

S5 Table Distribution of Biological Parameters used to define Metabolic Syndrome (MetS). (DOC)

S6 Table Median Sex Hormone Binding Globulin (SHBG) Levels by rs1799941 genotype in Metabolic Syndrome Controls and Cases. (DOC)

S7 Table Mean Sex Hormone Binding Globulin (SHBG) Levels by rs1799941 genotype in Metabolic Syndrome Controls and Cases. (DOC)

S8 Table Distribution of Age, Gender, and Body Mass Index (BMI) by rs1799941 genotype. (DOC)

S9 Table Effect of Metabolic Syndrome Case/Control Status on Median SHBG Levels. (DOC)

S10 Table Effect of Metabolic Syndrome Case/Control Status on Mean SHBG Levels. (DOC)
