# Supplementary figures and images for: SHBG Gene Polymorphism (rs1799941) Associates with Metabolic Syndrome in Children and Adolescents
Source: PLoS One. 2015 Feb 3;10(2):e0116915. doi: 10.1371/journal.pone.0116915 (PMC4380117; doi:10.1371/journal.pone.0116915)

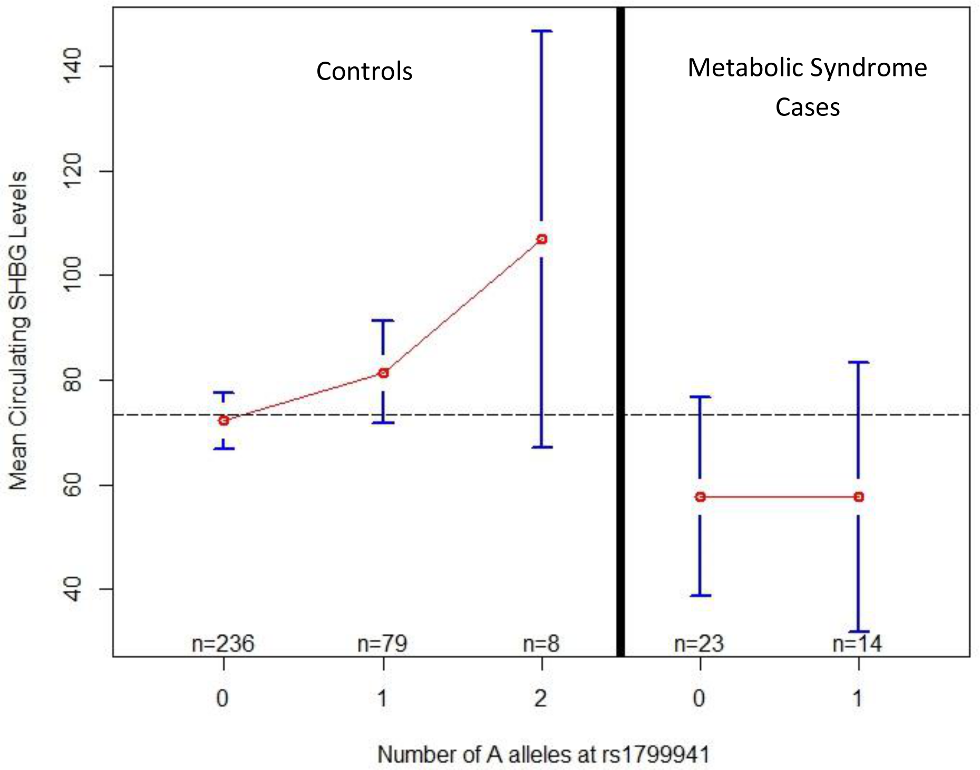

Supplement: S1 Fig — presents the relationship between mean SHBG levels and rs1799941 coded additively for increasing numbers of the minor allele (A allele). Dashed line represents overall mean SHBG level (73.8 nmol/l) in full cohort. Red circles represent genotypic means, and error bars represent 95% Confidence Intervals of associated genotypic means. Connecting red lines illustrate trend in mean SHBG levels by rs179941 genotype. (TIF) [file pone.0116915.s001.tif]

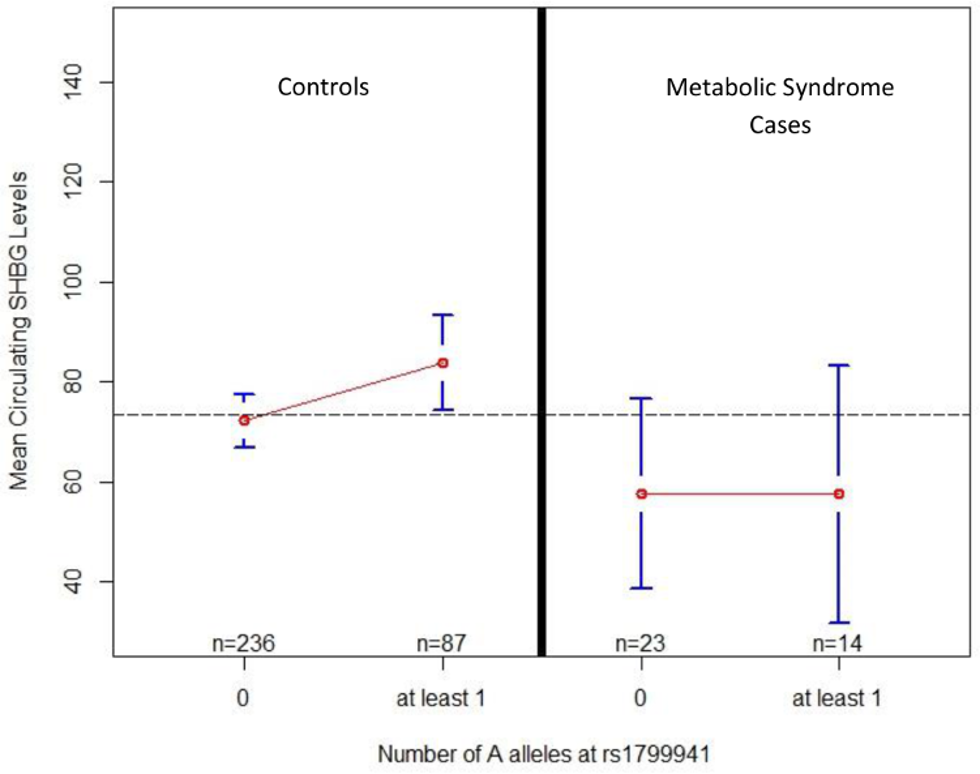

Supplement: S2 Fig — illustrates the relationship between mean SHBG levels and rs1799941 genotype coded dominantly for increasing numbers of the minor allele (A allele). Dashed line represents overall mean SHBG level (73.8 nmol/l) in full cohort. Red circles represent genotypic means, and error bars represent 95% Confidence Intervals of associated genotypic means. Connecting red lines illustrate trend in mean SHBG levels by rs179941 genotype. (TIF) [file pone.0116915.s002.tif]
