# Supplementary material for: SHBG Gene Polymorphism (rs1799941) Associates with Metabolic Syndrome in Children and Adolescents
Source: PLoS One. 2015 Feb 3;10(2):e0116915. doi: 10.1371/journal.pone.0116915 (PMC4380117; doi:10.1371/journal.pone.0116915)
Supplement: S1 Table — (DOC) [file pone.0116915.s003.doc]

Table S1. Genotyped single nucleotide polymorphisms (SNPs).

| Chromosome | Gene | SNP | Alleles^1^ |
| --- | --- | --- | --- |
| 8 | LPL | rs328 | S/X^2^ |
| 9 | ABCA1 | rs1800977 | C/T |
| 15 | LIPC | rs1800588 | C/T |
| 16 | CETP | rs708272 | B1/B2^3^ |
| 17 | SHBG | rs1799941 | G/A |
| 17 | SHBG | rs6257 | T/C |

^1^ Alleles: Major Allele / Minor Allele

^2^: rs328 is an LPL gene stop codon SNP, S (serine amino acid) 447Ter (termination codon). LPL S447X (rs328) involves a C→G change at nucleotide 1595 of the LPL gene, which leads to a change in amino acid 447 from a serine (S) to a stop codon (X). Heterozygotes for the X447 allele are displayed as SX and S447 homozygotes are listed as SS.

^3^: rs708272 is a silent base change SNP; a base substitution from G (B1) to A (B2) in intron 1 of the CETP gene leads to 3 variants, B1B1, B1B2 or B2B2 at Taq1B site (5454G>A).
