# Supplementary material for: SHBG Gene Polymorphism (rs1799941) Associates with Metabolic Syndrome in Children and Adolescents
Source: PLoS One. 2015 Feb 3;10(2):e0116915. doi: 10.1371/journal.pone.0116915 (PMC4380117; doi:10.1371/journal.pone.0116915)
Supplement: S2 Table — (DOC) [file pone.0116915.s004.doc]

Table S2. Genotype distribution and Hardy Weinberg Equilibrium (HWE) Analysis for all SNPs

|  | | | Overall | | Cases | | Controls | |
| --- | --- | --- | --- | --- | --- | --- | --- | --- |
| Gene | SNP | Genotype Categories | Genotype Counts (%) | HWE  P^1^ | Genotype Counts (%) | HWE P^1^ | Genotype Counts (%) | HWE P^1^ |
| ABCA1 | rs1800977 | CC | 163 (45.3) | 0.27 | 13 (35.1)  20 (54.1)  4 (10.8) | 0.49 | 150 (46.4)  146 (45.2)  27 (8.4) | 0.36 |
|  |  | CT | 166 (46.1) |  |  |  |  |  |
|  |  | TT | 31 (8.6) |  |  |  |  |  |
| LPL | rs328 | SS | 291 (80.8) | 0.06 | 30 (81.1)  7 (18.9) | 1.00 | 261 (80.8)  62 (19.2) | 0.06 |
|  |  | SX | 69 (19.2) |  |  |  |  |  |
| CETP | rs708272 | B1B1 | 108 (30.0) | 0.60 | 13 (35.1)  20 (54.1)  4 (10.8) | 0.50 | 95 (29.4)  154 (47.7)  74 (22.9) | 0.44 |
|  |  | B1B2 | 174 (48.3) |  |  |  |  |  |
|  |  | B2B2 | 78 (21.7) |  |  |  |  |  |
| LIPC | rs1800588 | CC | 244 (67.8) | 1.00 | 24 (64.9)  12 (32.4)  1 (2.7) | 1.00 | 220 (68.1)  93 (28.8)  10 (3.1) | 1.00 |
|  |  | CT | 105 (29.2) |  |  |  |  |  |
|  |  | TT | 11 (3.1) |  |  |  |  |  |
| SHBG | rs1799941 | GG | 259 (71.9) | 1.00 | 23 (62.2)  14 (37.8)  0 (0.0) | 0.31 | 236 (73.0)  79 (24.5)  8 (2.5) | 0.66 |
|  |  | AG | 93 (25.8) |  |  |  |  |  |
|  |  | AA | 8 (2.2) |  |  |  |  |  |
|  | rs6257 | TT | 237 (65.8) | 0.01 | 24 (64.9)  12 (32.4)  1 (2.7) | 1.00 | 213 (65.9)  107 (33.1)  3 (1.0) | 0.01 |
|  |  | CT | 119 (33.1) |  |  |  |  |  |
|  |  | CC | 4 (1.1) |  |  |  |  |  |

^1^Hardy Weinberg Equilibrium test p-value is presented.
