# Supplementary material for: SHBG Gene Polymorphism (rs1799941) Associates with Metabolic Syndrome in Children and Adolescents
Source: PLoS One. 2015 Feb 3;10(2):e0116915. doi: 10.1371/journal.pone.0116915 (PMC4380117; doi:10.1371/journal.pone.0116915)
Supplement: S3 Table — (DOC) [file pone.0116915.s005.doc]

Table S3. Dominantly Coded Genotype Distribution for all SNPs in Metabolic Syndrome Cases and Controls.

| Gene | SNP | Genotype Categories^1^ | Genotype Counts (%) | Genotype Counts (%) | Genotype Counts (%) |
| --- | --- | --- | --- | --- | --- |
| ABCA1 | rs1800977 | CC | 163 (45.3) | 13 (35.1)  24 (64.9) | 150 (46.4)  173 (53.6) |
|  |  | CT/TT | 197(54.7) |  |  |
| LPL | rs328* | SS | 291 (80.8) | 30 (81.1)  7 (18.9) | 261 (80.8)  62 (19.2) |
|  |  | SX | 69 (19.2) |  |  |
| CETP | rs708272 | B1B1 | 108 (30.0) | 13 (35.1)  24 (64.9) | 95 (29.4)  228 (70.6) |
|  |  | B1B2/B2B2 | 252 (70.0) |  |  |
| LIPC | rs1800588 | CC | 244 (67.8) | 24 (64.9)  13 (35.1) | 220 (68.1)  103 (31.9) |
|  |  | CT/TT | 116 (32.2) |  |  |
| SHBG | rs1799941 | GG | 259 (71.9) | 27 (73.0)  10 (27.0) | 232 (71.8)  91 (28.2) |
|  |  | AG/AA | 101 (28.1) |  |  |
|  | rs6257 | TT | 237 (65.8) | 21 (56.8)  16 (43.2) | 216 (66.9)  107 (33.1) |
|  |  | CT/CC | 123 (34.2) |  |  |

*Note: Genotype categories above reflect dominant coding in reference to the minor allele, unless otherwise noted.*

^1^Genotype Category definitions: MM = homozygote major, Mm/mm = combined heterozygote and homozygote minor; M=major allele, m=minor allele.

*Genotype counts were not coded dominantly as there were no individuals with homozygous minor genotype present in the study cohort.
