# Supplementary material for: SHBG Gene Polymorphism (rs1799941) Associates with Metabolic Syndrome in Children and Adolescents
Source: PLoS One. 2015 Feb 3;10(2):e0116915. doi: 10.1371/journal.pone.0116915 (PMC4380117; doi:10.1371/journal.pone.0116915)
Supplement: S4 Table — (DOC) [file pone.0116915.s006.doc]

Table S4. Definition of dichotomized cardio-metabolic traits included in adjusted logistic regression models for the evaluation of the association between rs1799941 and MetS

|  | Dichotomization Scheme for Cardio-Metabolic Traits | |
| --- | --- | --- |
| Cardio-Metabolic Trait included in MetS definition | Absence (0) | Presence (1) |
| High Triglycerides (High TG) | <75^th^ percentile | ≥ 75^th^ percentile |
| Low High-density lipoprotein cholesterol ( Low HDL-c) | >25^th^ percentile | ≤ 25^th^ percentile |
| Obese/Overweight based on age and gender specific international Body Mass Index (BMI) cut off points | BMI < age and gender specific international BMI cut off point for overweight or obese status | BMI ≥ age and gender specific international BMI cut off point for overweight or obese status |
| Increased Waist Circumference | < 90^th^ percentile of locally representative sample | ≥ 90th percentile of locally representative sample |
| Elevated Blood Pressure (Elevated BP) | <95^th^ percentile | ≥ 95^th^ percentile |
| Insulin Resistance | HOMA-IR < 3.16 | HOMA-IR ≥ 3.16 |
| Increased Waist Circumference | < 95^th^ percentile | ≥ 95^th^ percentile |

*Note: Numbers in () above indicate the coding scheme applied to the cardio-metabolic traits when they were included in logistic regression analyses. The absence of the specified trait (coded 0) was used as the referent group.*
