# Supplementary material for: SHBG Gene Polymorphism (rs1799941) Associates with Metabolic Syndrome in Children and Adolescents
Source: PLoS One. 2015 Feb 3;10(2):e0116915. doi: 10.1371/journal.pone.0116915 (PMC4380117; doi:10.1371/journal.pone.0116915)
Supplement: S5 Table — (DOC) [file pone.0116915.s007.doc]

Table S5. Distribution of Biological Parameters used to define Metabolic Syndrome (MetS)

|  | Distribution of Metabolic Syndrome Criteria^1^ | |
| --- | --- | --- |
| Metabolic Syndrome Criteria | MetS Cases^1^ n=37 | Controls n=323 |
| Elevated diastolic or systolic blood pressure  ( ≥ 95th percentile) | 19 (51.4%) | 58 (18.0 %) |
| Overweight or obese (BMI ≥ age and gender specific international BMI cut off point) | 22 (59.5%) | 20 (6.19 %) |
| Elevated triglycerides ( ≥ 75th percentile) | 36 (97.3%) | 57 (17.7%) |
| Low HDL cholesterol (≤ 25th percentile) | 31 (83.8%) | 40 (12.4 %) |
| Elevated Glucose ( ≥ 100mg/dL) | 0 (0%) | 2 (0.62 %) |
| Insulin Resistance (HOMA-IR ≥ 3.16) | 9 (24.3%) | 31 (9.60 %) |
| Increased waist circumference  (≥ 90th percentile, ethnicity specific) | 17 (46.0%) | 19 (5.88 %) |
| Raised triglycerides  (≥ 150 mg/dL) | 6 (16.22%) | 5 (1.55%) |
| Reduced HDL-cholesterol  (≤40mg/dL for boys, ≤ 50mg/dL for girls) | 24 (64.9%) | 122 (37.77%) |
| Raised blood pressure  (systolic ≥ 130 mm Hg, diastolic ≥85 mm Hg) | 6 (6.2%) | 29 (8.71 %) |

*Note: Number of cases that fit specified metabolic syndrome criteria shown above do not represent unique individuals as MetS case definition required the presence of more than one cardio-metabolic trait.*

^1^Numberof individuals meeting specified MetS criterion, (corresponding percentage)
