# Supplementary material for: SHBG Gene Polymorphism (rs1799941) Associates with Metabolic Syndrome in Children and Adolescents
Source: PLoS One. 2015 Feb 3;10(2):e0116915. doi: 10.1371/journal.pone.0116915 (PMC4380117; doi:10.1371/journal.pone.0116915)
Supplement: S6 Table — (DOC) [file pone.0116915.s008.doc]

Table S6. Median Sex Hormone Binding Globulin (SHBG) Levels by rs1799941 genotype in Metabolic Syndrome Controls and Cases

|  | | Controls, n=323 | | | Metabolic Syndrome Cases, n=37 | | |
| --- | --- | --- | --- | --- | --- | --- | --- |
|  |  | SHBG Levels | | | SHBG Levels | | |
| Genotyping Model | Number of minor alleles | N | Median | Interquartile Range | N | Median | Interquartile Range |
| Additive | 0 | 236 | 62 | [37.00 – 101.00] | 23 | 51 | [22.50 – 73.00] |
|  | 1 | 79 | 71 | [46.00 – 115.00] | 14 | 31 | [22.00 –108.00] |
|  | 2 | 8 | 99 | [70.50 – 153.00] | 0 | NA | NA |
|  | | | | | | | |
| Dominant | 0 | 236 | 62 | [37.00 – 101.00] | 23 | 51 | [22.50 – 73.00] |
|  | ≥1 | 87 | 77 | [46.50 – 119.00] | 14 | 31 | [22.00 –108.00] |
