# Supplementary material for: SHBG Gene Polymorphism (rs1799941) Associates with Metabolic Syndrome in Children and Adolescents
Source: PLoS One. 2015 Feb 3;10(2):e0116915. doi: 10.1371/journal.pone.0116915 (PMC4380117; doi:10.1371/journal.pone.0116915)
Supplement: S7 Table — (DOC) [file pone.0116915.s009.doc]

Table S7. Mean Sex Hormone Binding Globulin (SHBG) Levels by rs1799941 genotype in Metabolic Syndrome Controls and Cases

|  | | Controls n=323 | | | Metabolic Syndrome Cases n=37 | | |
| --- | --- | --- | --- | --- | --- | --- | --- |
|  |  | SHBG Levels | | | SHBG Levels | | |
| Genotyping Model | Number of minor alleles | N | Mean | SE^1^ | N | Mean | SE^1^ |
| Additive | 0 | 236 | 72.18 | 2.71 | 23 | 57.70 | 9.17 |
|  | 1 | 79 | 81.48 | 4.96 | 14 | 57.57 | 11.91 |
|  | 2 | 8 | 106.88 | 16.80 | 0 | NA | NA |
|  | | | | | | | |
| Dominant | 0 | 236 | 72.18 | 2.71 | 23 | 57.70 | 9.17 |
|  | ≥1 | 87 | 83.82 | 4.80 | 14 | 57.57 | 11.91 |

^1^SE: Standard Error of the Mean
