# Supplementary material for: SHBG Gene Polymorphism (rs1799941) Associates with Metabolic Syndrome in Children and Adolescents
Source: PLoS One. 2015 Feb 3;10(2):e0116915. doi: 10.1371/journal.pone.0116915 (PMC4380117; doi:10.1371/journal.pone.0116915)
Supplement: S8 Table — (DOC) [file pone.0116915.s010.doc]

Table S8. Distribution of Age, Gender, and Body Mass Index (BMI) by rs1799941 genotype

| Demographic Variable | rs1799941 genotype groups | | | | | P-Value |
| --- | --- | --- | --- | --- | --- | --- |
|  | GG | AG | | | AA |  |
| Age^1^ | 12.97 [0.21] | | 13.17 [0.35] | 11.25 [0.88] | | 0.363 |
| Gender^2^ | 50.2% | | 59.1% | 62.5% | | 0.119^4^ |
| Body Mass Index (BMI)^3^ | 18.9 [0.23] | | 19.1 [0.45] | 16.7 [0.89] | | 0.216 |

*Note:* P-values shown above are from the non-parametric Kruskal-Wallis equality of populations rank test unless otherwise indicated.

^1^ Mean [Standard Error] of age measured in years

^2^ Percentage of indicated Metabolic Syndrome subset (i.e. cases or controls) that is female

^3^Mean [Standard Error] of BMI is presented

^4^P-value presented is from the Non-Parametric Trend test (increasing trend)
