# Supplementary material for: SHBG Gene Polymorphism (rs1799941) Associates with Metabolic Syndrome in Children and Adolescents
Source: PLoS One. 2015 Feb 3;10(2):e0116915. doi: 10.1371/journal.pone.0116915 (PMC4380117; doi:10.1371/journal.pone.0116915)
Supplement: S9 Table — (DOC) [file pone.0116915.s011.doc]

Table S9. Effect of Metabolic Syndrome Case/Control Status on Median SHBG Levels

| Median SHBG Predictor Variable | Coef.^1^ | SE^2^ | P-Value | 95% Confidence Interval |
| --- | --- | --- | --- | --- |
| MetS_Status^3^ | -28.00 | 4.71 | 6.48E-09 | -37.25699 – -18.74301 |
| AGE | -20.63 | 1.38 | 1.59E-39 | -23.33882 – -17.91118 |
| GENDER^4^ | -112.13 | 11.52 | 5.25E-20 | -134.7767 – -89.47327 |
| AGE x GENDER^5^ | 8.13 | 0.86 | 5.03E-19 | 6.432543 – 9.817457 |

^1^Change in median SHBG per one unit increase in the indicated predictor variable, holding all others constant

^2^Standard Error

^3^Controls were coded as 0, MetS cases were coded as 1

^4^Males were coded as 0, Females were coded as 1

^5^Multiplicative age by gender interaction term; calculated by multiplying the age and gender variables together
