# Supplementary material for: SHBG Gene Polymorphism (rs1799941) Associates with Metabolic Syndrome in Children and Adolescents
Source: PLoS One. 2015 Feb 3;10(2):e0116915. doi: 10.1371/journal.pone.0116915 (PMC4380117; doi:10.1371/journal.pone.0116915)
Supplement: S10 Table — (DOC) [file pone.0116915.s012.doc]

Table S10. Effect of Metabolic Syndrome Case/Control Status on Mean SHBG Levels

| Mean SHBG Predictor Variable | Beta^1^ | SE^2^ | P-Value | 95% Confidence Interval |
| --- | --- | --- | --- | --- |
| MetS_Status^3^ | -27.40 | 5.26 | 3.20E-07 | -37.74158 – -17.05824 |
| AGE | -18.97 | 1.52 | 7.37E-30 | -21.95625 – -15.97532 |
| GENDER^4^ | -92.02 | 12.72 | 2.92E-12 | -117.0373 – -67.00085 |
| AGE x GENDER^5^ | 6.82 | 0.95 | 4.08E-12 | 4.950134 – 8.683913 |

^1^Change in mean SHBG per one unit increase in the indicated predictor variable, holding all others constant

^2^Standard Error

^3^Controls were coded as 0, MetS cases were coded as 1

^4^Males were coded as 0, Females were coded as 1

^5^Multiplicative age by gender interaction term; calculated by multiplying the age and gender variables together
